# Updates in the Treatment of Knee Osteoarthritis

**DOI:** 10.1055/s-0044-1786351

**Published:** 2024-06-22

**Authors:** Diego Pontes de Carvalho Pires, Felipe Alves do Monte, Leonardo Freire Monteiro, Francisco Rafael do Couto Soares, José Leonardo Rocha de Faria

**Affiliations:** 1 Instituto de Coluna e Ortopedia Especializada (InCore), Recife, PE, Brasil; 2 Departamento de Ortopedia e Traumatologia, Hospital da Restauração Governador Paulo Guerra, Recife, PE, Brasil; 3 Ortoclínica, Recife, PE, Brasil; 4 Serviço de Traumatologia e Ortopedia, Hospital Metropolitano Norte Miguel Arraes (HMA), Recife, PE, Brasil; 5 Serviço de Traumatologia e Ortopedia, Instituto de Medicina Integral Professor Fernando Figueira (IMIP), Recife, PE, Brasil; 6 Instituto Nacional de Traumatologia e Ortopedia (INTO), Rio de Janeiro, RJ, Brasil

**Keywords:** arthroscopy, articular cartilage, biomarkers, physical exercise, intra-articular infiltrations, knee, osteoarthritis, physical exercise

## Abstract

Knee osteoarthritis (OA) is an inflammatory and degenerative condition resulting in articular cartilage destruction and functional loss. Its prevalence has grown considerably due to increased life expectancy and obesity, and its diagnosis relies on evaluation, medical examination, and confirmation by supplementary radiographic images. Knee OA is multifactorial and influenced by several local, systemic, and external aspects. In addition, its progress and therapeutic responses highly depend on the characteristics of each subject. The initial recommendation is drug treatment and alternative therapies to improve quality of life. However, if these treatments are unsuccessful, one must consider surgical treatment. Surgical options include arthroscopies, osteotomies, and partial and total arthroplasties, while non-surgical treatments include medications and alternative therapies such as infiltrations, acupuncture, and physical exercise. It is worth highlighting that biomarkers can be a significant strategy for early disease detection, assessment of disease activity, prediction of prognosis, and monitoring a better response to therapy. Nevertheless, this topic must be the focus of further research to confirm its findings.

## Introduction

Osteoarthritis (OA) causes cartilage degradation, bone remodeling, osteophyte formation, and synovial inflammation, resulting in pain, stiffness, edema, and loss of joint function. It is a significant cause of disability among adults and older subjects. 1 2 Populational aging and the progressively higher prevalence of obesity are increasing the incidence of this disease. 2

The most common knee disease, OA may result in repeated joint effusion and functional limitation. 3 A slow and steady understanding of the pathophysiology of knee OA has been witnessed in recent decades. However, gaps regarding its pathogenesis remain, negatively impacting drug development and innovation. The lack of new medications and modifying drugs for knee OA treatment highlights the need for lifestyle changes to delay surgical intervention. 4 Furthermore, medical societies recommend low-impact physical activity, and surgical interventions may have conflicting outcomes. 5 6 7

Additionally, the use of biological therapies has been increasing as alternatives to OA treatment, particularly intra-articular (IA) infiltrations. Hyaluronic acid (HA) is widely known as a safe conservative treatment for OA in the knee and other joints. 8 It provides pain relief, stimulates synovial fluid production, and cushions and lubricates joint surfaces. 9

A growing body of scientific literature supports the use of IA infiltrations with HA for knee OA over placebo and other therapies. Acharya et al. 10 analyzed the effectiveness of IA with HA regarding pain, functional capacity, and quality of life in patients with early primary knee OA. These authors 10 reported short-term beneficial effects (up to one year), observing a decreasing trend in pain intensity and a progressive improvement in physical functioning and health-related quality of life after a single intervention.

Knee OA is multifactorial and influenced by several local, systemic, and external aspects. In addition, its progress and therapeutic responses highly depend on the characteristics of each subject. To better understand this condition, the present study aimed to gather information about its pathophysiology, clinical diagnosis, and updates on its treatment.

## Pathophysiology of Knee Osteoarthritis

Osteoarthritis results from complex biological processes affecting cartilage, bone, ligaments, synovium, meniscus, periarticular fat, and muscles. It usually presents with joint space narrowing (JSN) due to articular cartilage loss, meniscal degeneration, and bone changes, including subchondral bone sclerosis and osteophytes. 11 12

The biomechanical environment can influence OA development. Varus alignment shifts the load medially, increasing the risk of knee OA in the medial compartment, while valgus alignment shifts the load laterally, triggering OA in the lateral compartment. Additionally, previous knee trauma increases the risk of OA by 3.86 times. 13

Mechanical overload often accounts for starting the process of cartilage damage, which later evolves into inflammation, causing joint degradation. The primary precursors of this inflammation process include interleukin-1 (IL-1) and tumor necrosis factor (TNF). These molecules maximize the expression of metalloproteinases and nitric oxide, the main catabolic agents synthesized by chondrocytes in response to injury. 14 Therefore, OA is an inflammatory disease of the entire synovial joint, comprising the mechanical and structural degeneration of the articular cartilage and triggering functional changes in the entire joint. 4

The development of OA is slow, and the pain can be progressive; 12 OA is a major cause of pain and disability, directly affecting quality of life. 2 It may result from several factors, including age, body weight disorders, knee joint overload, prolonged hyperflexion, and axis deviations. 3

According to Primorac et al., 15 the prevalence of knee OA is higher in adults aged ≥ 60 years, representing one of the most significant causes of disability among elderly subjects. In addition, the risk is higher in obese people (body mass index [BMI] ≥ 30 kg/m ^2^ ) than in non-obese people (19.7% versus 10.9% respectively).

## Clinical Diagnosis and Classification Systems

The diagnosis is initially clinical, based on symptoms of pain, stiffness, and functional limitations, as well as a thorough physical examination with crepitus, pain, restricted mobility, joint sensitivity, and bone enlargement observation. 2 Weight-bearing knee radiographs help confirm the diagnosis and enable the classification of knee OA and the observation of structural damage. In addition, they improve specificity when osteophytes or JSN are present. 16

Magnetic resonance imaging (MRI) reveals the effects of knee OA on the cartilage, meniscus, synovial membrane, and subchondral bone. Moreover, a tool developed by Cho et al. 17 uses infrared immunoliposomes against type-II collagen antibodies for early OA diagnosis and treatment.

Classifications are critical in knee surgery since effective classification systems can guide prognostic estimates. The recent publication by Pires et al. 18 , “Classifications and flowcharts in knee surgery,” is a Brazilian reference study that combines several classifications, flowcharts, and questionnaires to aid in the diagnosis and decision-making by specialists.

Following the classifications for degenerative lesions, 18 the most common method for radiographic definition is the Kellgren-Lawrence classification system. The overall joint score classifies OA as follows: grade 0 represents no pathological findings; grade 1 indicates potential JSN and edge osteophytes; grade 2 indicates potential JSN and definitive osteophytes; grade 3 establishes the presence of JSN, multiple osteophytes, some subchondral sclerosis, and potential bone contour deformity; and grade 4 denotes notable JSN, severe subchondral sclerosis, definitive bone contour deformity, and the presence of large osteophytes. 19

## Clinical Examination and Imaging

About 40% of the population aged over 65 years has symptomatic knee OA. Early detection and intervention are paramount to reduce morbidity and disability, resulting in better self-reliance. Radiography is the first investigation of choice for OA patients presenting with knee pain. Community-based studies 19 have shown that 40% to 80% of subjects with OA and radiographic knee changes are symptomatic, and that severe radiographic findings are associated with reports of pain that is more intense. However, there is a high degree of discordance between clinical and radiographic findings, and radiological OA classification is inaccurate in the early stages. The multifactorial origin of pain may explain this discrepancy between pain and radiographic findings. 19

The discrepancy between the physical and imaging examinations, especially concerning symptoms, can be explained by the propensity of some OA patients to develop sensitized central nociceptive circuits that increase pain during various states of aggression to peripheral tissues. 20 21 This abnormality, known as central sensitization, is a maladaptive nociceptive process involving complex neuroplastic changes amplifying pain at multiple levels of the neuraxis. 20 Since central sensitization correlates with the activation of neural circuits implicated in the descending facilitation of pain and, as such, is a risk factor for the development and maintenance of chronic pain, it is essential to identify which patients present abnormal responses to relevant painful stimuli.

### Biomarkers

An accurate and reliable biomarker must present specificity for a given condition and reflect the actual activity of the disease, monitor the changes achieved with therapy, and predict the prognosis. 20 Thus, most biochemical markers of OA characterize cartilage renewal. The most commonly investigated markers include the following: for extracellular matrix (ECM) degradation – urinary C-telopeptide fragments of type-II collagen (uCTX-II), Coll2–1, C2C, C2M, Coll2–1NO2, cartilage oligomeric matrix protein (COMP), aggrecan epitopes (ARGS, TEGE, FFGV), fibulin-3 epitopes (Fib3–1, Fib3–2, Fib3–3) etc.; and, for ECM synthesis, PIIANP, PIIBNP, CPII, CS846 etc. 21 The uCTX-II) is one of the best-known biochemical markers of OA, achieving a superior predictive profile compared to others.

According to the literature, several conditions can affect biomarker levels. Some evidence suggests that physical activity, hormonal levels, medications, and the sex of the patient may result in biomarker level fluctuations. Tanishi et al. 22 observed that uCTX-II levels differed significantly between men and women and in premenopausal and post-menopausal women.

Due to its ability to distinguish between healthy subjects and OA patients, another potential biochemical marker widely reported in the literature is COMP, 23 which also has some potential prognostic capabilities. Although some studies have reported conflicting results, a meta-analysis 23 showed that these biochemical markers (that is, COMP and CTX-II) may be effective to diagnose OA, assess prognosis progression, and differentiate between healthy subjects and those with OA.

The emergence of these biomarkers is relatively recent, and the best strategies for their application remain under study, in terms of technology and medical research, with the goal of developing reliable detection methods.

### Treatments

The first-line treatment for knee OA does not involve surgical interventions, and management may be multimodal. However, if these therapies are unsuccessful, one must consider surgical treatment. Although OA has no cure, several surgical and non-surgical treatment options are available to help manage pain and maintain the health of the affected patients. 12

The following section will discuss the most widespread treatments according to their current level of recommendation.

## Materials and Methods

Search Strategy

A bibliographical survey was performed in PubMed, MEDLINE, and Embase for studies published until February 20, 2023. The descriptors included *Updates* ; *Knee* *Osteoarthrosis* , and *Treatment* , and the filters included *Randomized Controlled Trial* , *Randomized Clinical Trial* , *Meta-Analysis* , *Systematic Reviews* , and *Cohort* .

### Eligibility and Selection Criteria

The retrieved studies met the following inclusion criteria:

- Studies related to the proposed topic;
- Studies with level of evidence of I to III;
- Articles in English, Spanish, and Portuguese;
- Research carried out on humans;
- Articles available in full versions.

The exclusion criterium was the following:

- Studies with a low level of evidence, such as a simple case reports.

## Results

### Identification of Studies and Characteristics

Following the search strategy, we found 41 studies with the aforementioned descriptors. These studies underwent a new assessment regarding their design, relevance, study type filters, and inclusion criteria. This assessment resulted in eight studies available in full and included according to category and type of treatment.

## Surgical Treatments

### Arthroscopy

**Fig. 1:**
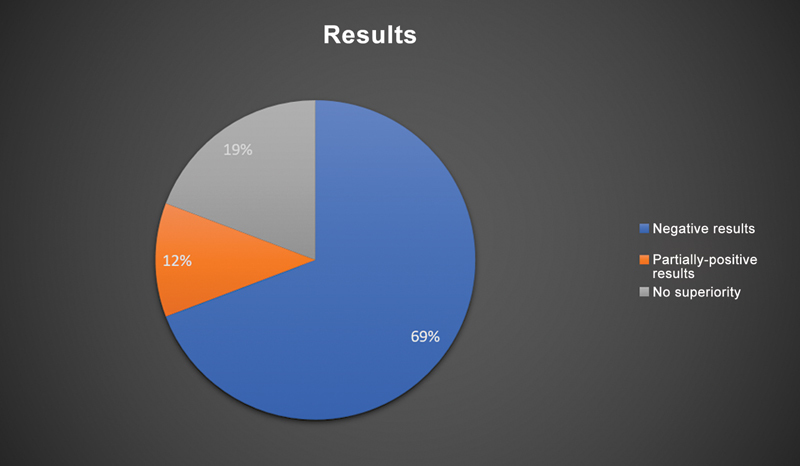
Graphical representation of the outcomes of studies assessing arthroscopy for the treatment of knee degenerative disease.

**Fig. 2:**
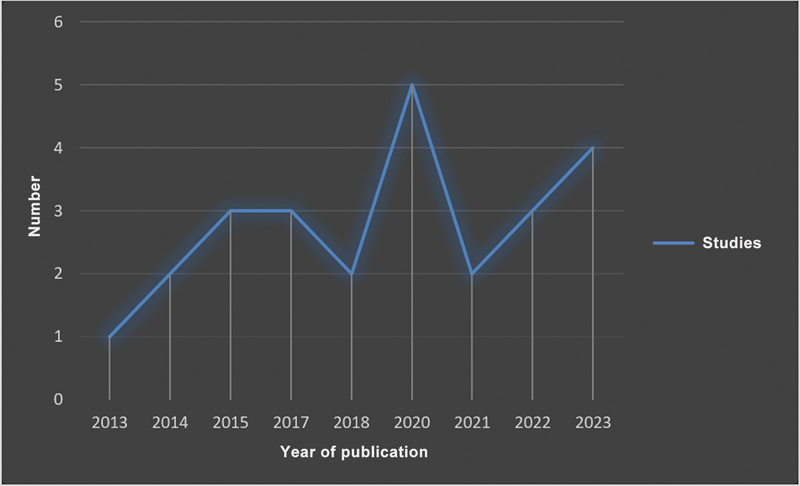
Temporal description of the literature included by year of publication.

Arthroscopy removes debris and crystals released in the joint cavity affected by OA, regularizing the joint surface. 24 A literature survey retrieved 26 systematic reviews and meta-analyses describing the results of 6,418 patients with a mean age of 47 ± 19 years ( Figs. 1 and 2 ).

Arthroscopic surgery for degenerative knee disease has been the focus of several recently published randomized clinical trials (RCTs), high-quality systematic reviews, and meta-analyses. Although pioneering studies have demonstrated that minimally-invasive arthroscopy had promising results, their strength was insufficient for a recommendation. Current clinical studies comparing arthroscopic joint debridement with placebo surgery or conservative treatment have demonstrated that arthroscopy is no longer effective and has no recommendation. 10 11 12 13 14 15 16 17 25

A recent systematic review 11 published in the *British Medical Journal* strongly recommended against arthroscopy in patients with degenerative knee disease, emphasizing the importance of fully utilizing appropriate conservative treatment options. In particular, the recommendations directed clinicians to maximize exercise therapy, as the results presented in RCTs demonstrate that arthroscopic surgeries are not superior to complete physiotherapy protocols. 11 Another systematic review of eight RCTs evaluating arthroscopic surgery in degenerative meniscal injuries found moderate evidence suggesting no clinical benefit of arthroscopic meniscal debridement for degenerative meniscal tears compared to non-surgical or sham treatments in middle-aged OA patients. 10 26 27 28

In a prospective cohort, Navarro et al. 29 aimed to ascertain whether knee arthroscopy to treat meniscal damage by OA delayed knee replacement compared with physiotherapy alone. The main findings were that the cumulative incidence of knee replacement was modest but significantly higher for subjects undergoing arthroscopy than physical therapy alone (risk ratio: 1.30; 95% confidence interval: 1. 17–1.44; *p* < 0.001). The authors 29 concluded that, for patients with meniscal damage complicated by OA, those who underwent arthroscopy were 30% more likely to have partial or total knee replacement surgery at any point during follow-up compared with those undergoing physical therapy alone.

#### Indications

For all these reasons, many researchers limit the indications for arthroscopy. The technique still presents advantages in the early stages of OA, in which movement limitation is mechanical, and there are free articular bodies. However, physical therapy must accompany arthroscopy. 26

For Zhao et al., 27 arthroscopy presents no advantages over the several surgical options due to the weak and scarce evidence supporting its application. Likewise, recent studies suggest that arthroscopy may not be beneficial for patients with chronic degenerative meniscal tears, emphasizing the importance of conservative follow-up for the management of knee OA. 28

The small and inconsequential benefit observed in interventions including arthroscopy for the degenerated knee is time-limited and absent within one to two years after surgery. Knee arthroscopy is associated with harm. Together, these findings do not support arthroscopic surgery for middle-aged or elderly patients with knee pain with or without signs of OA.

### Arthroplasty

Arthroplasty is a surgical option to treat patients with extreme cases of knee joint degeneration; it can be total (total knee replacement, TKR) or unicompartmental (partial knee replacement, PKR), depending on the degree of OA. 30

**Fig. 3:**
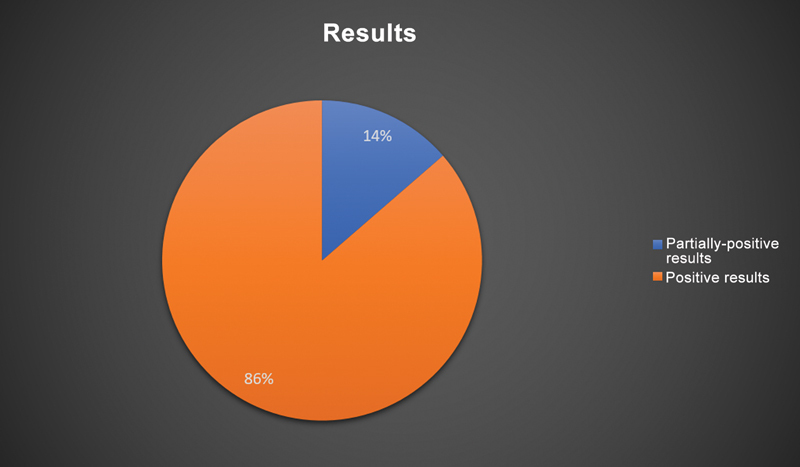
Graphical representation of the outcomes of studies assessing (total or partial) arthroplasty for the treatment of knee degenerative disease.

**Fig. 4:**
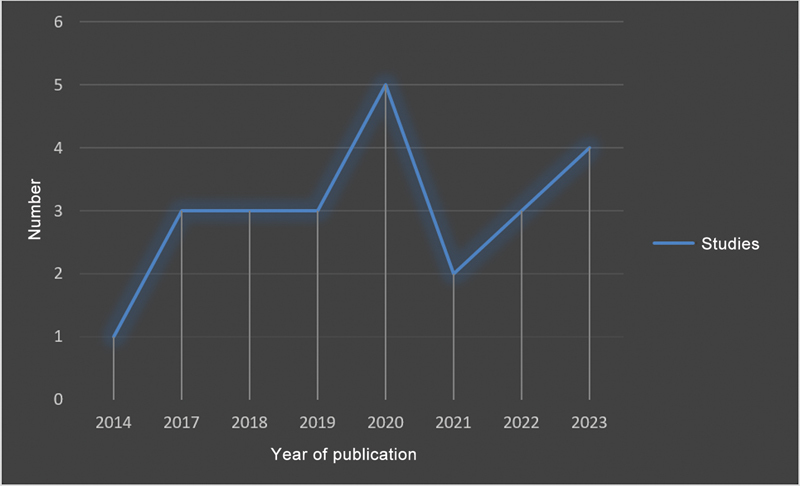
Temporal description of the literature included by year of publication.

In the literature survey, we found 22 systematic reviews and meta-analyses describing the outcomes of 14,095 patients with a mean age of 58 ± 21 years ( Figs. 3 and 4 ).

For OA limited to a single compartment, PKR or unloading osteotomy may be an option. Their recommendation includes young, active patients because of the risks and limited durability of TKR. In contrast, TKR is a common, safe method in elderly patients with advanced knee OA and the gold-standard surgical treatment for advanced cases. 30

An RCT 31 demonstrated that TKR is superior to non-surgical treatment alone in relieving pain in patients with moderate to severe knee OA and in improving function and quality of life after one year. However, both groups presented clinically relevant improvements, and patients undergoing TKR had more severe side effects. Furthermore, resection of the interpatellar fat pad during TKR is the subject of ongoing debate, with no clear consensus. 31

#### Indications

The indication for TKR includes patients with advanced joint degeneration process and subjects older than 60 years. It is also the gold-standard treatment in this last group of patients. The indication for PKR includes young, active patients due to the risks and limited durability of TKR.

### Osteotomy

High tibial osteotomy (HTO) has been used for over sixty years as a clinical exercise. This technique, performed around the knee, aims to maintain a regular anatomical structure, achieve better functional recovery of the knee joint, attenuate joint softness, reduce the cartilage degeneration rate, and prevent or postpone joint replacement. 32 This technique relies on a controlled osteotomy, transferring the weight-bearing axis from the degenerate compartment to the healthy compartment to realign the lower limb. 33 As a result, this procedure interferes little with soft tissues and often has no harmful effects on the stability and mobility of the knee joint. 34

**Fig. 5:**
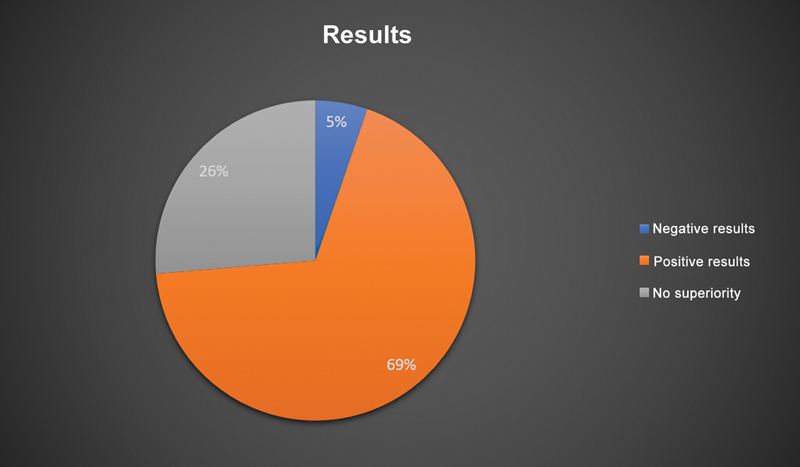
Graphical representation of the outcomes of studies assessing osteotomy for the treatment of knee degenerative disease.

**Fig. 6:**
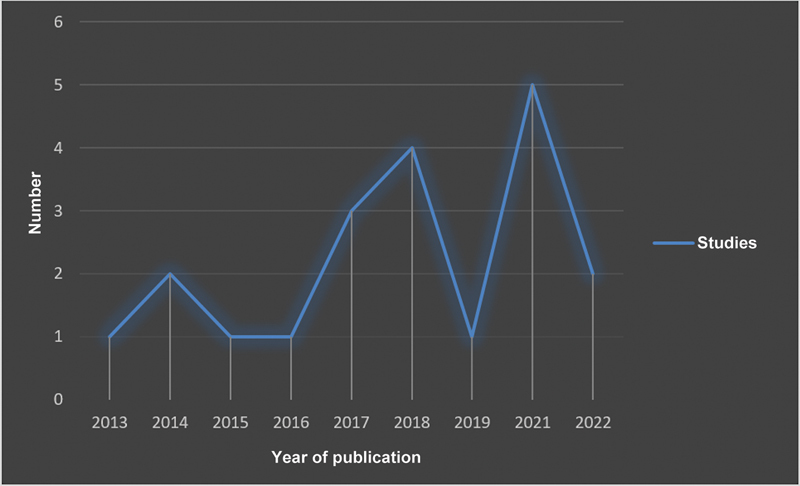
Temporal description of the literature included by year of publication.

In the literature survey, we found 19 systematic reviews and meta-analyses describing the outcomes of 8,412 patients with a mean age of 51 ± 7 years ( Figs. 5 and 6 ).

High tibial osteotomy comprises two main techniques: closing-wedge HTO (CWHTO) and opening-wedge OTA (OWHTO). 34 These procedures may occur proximal or distal to the knee joint. In general, if knee OA is in the medial compartment, HTO is most commonly performed by operating on the upper tibia. In OA affecting the lateral compartment, HTO is usually performed on the lower femur. However, one must always measure the distal lateral femoral angles and the medial proximal tibial angle to optimize the correct osteotomy location and whether it will be an addition or subtraction following the principles described by Paley and Tetsworth. 35

The indication for HTO includes young, active subjects, often under the age 50. Several factors may influence HTO outcomes. 36 Valeiy Evgenievich et al. 37 demonstrated a greater efficiency of HTO in the second stage of knee OA than in the third stage. These authors also noted that age and correction angle do not affect the outcome, but high BMI is associated with worse results and complications. The literature heterogeneously reports its imprecision, including lower functional outcomes, OA progression, and conversion to arthroplasty.

Lastly, HTO is an autonomous treatment, but the process of limb alignment intrinsically relates to the joint and stabilizing knee structures. Therefore, one must consider combining HTO with other osteotomies and arthroplasties. In addition, it may be a treatment strategy in procedures aiming to stabilize the knee and restore the cartilage.

#### Indications

High tibial osteotomy is suitable for young patients, especially those with OA and partially preserved cartilage. For knee biomechanics correction, the highest efficacy of HTO occurs in patients with second-stage knee OA compared to the third stage.

Proximal fibular osteotomy (PFO) is more suitable for patients with many complications and low surgical tolerance, as it can reduce operative time, intraoperative bleeding, hospitalization time, and postoperative complications, with some advantages.

## Non-surgical Treatments

### Pharmacological Therapies

Pharmacological therapy for OA is purely symptomatic and, in most cases, successfully assures the maintenance of the patient's mobility. It may include paracetamol, topical or oral non-steroidal anti-inflammatory drugs (NSAIDs), or intra-articular corticosteroids. Although paracetamol is a drug of considerable efficacy, safety, and low cost, the Osteoarthritis Research Society International (OARSI) guidelines recommend its administration in conservative doses and periods because of the increased risk of gastrointestinal and liver disorders. 38

In parallel, NSAIDs are the first-line pharmacological treatment for OA, with evidence proven by placebo-controlled trials. Topical NSAIDs often result in less gastrointestinal toxicity than oral NSAIDs. However, one must discuss alternatives to NSAIDs for patients who are susceptible or present risk factors for the adverse effects of the medication. It is worth highlighting that drugs should complement, not replace, the non-pharmacological treatment.

#### Indications

Patients with symptomatic OA.

##### Intra-articular Infiltrations

Biological therapies have been increasingly sought after as alternatives for OA treatment. Intra-articular infiltrations stand out as treatments approved by the Food and Drug Administration (FDA) and the European Medicines Agency (EMA). 39 These infiltrations enable the administration of a concentrated drug dosage and its distribution locally and throughout the joint. Thus, these drugs can access places that it would not with oral intake. Furthermore, IA infiltrations offer much better bioavailability and limit the systemic drug passage, reducing the risk of side effects. 39 40

#### Indications

Intra-articular infiltrations are an adjuvant therapy for OA treatment, especially in patients already under chronic systemic treatment, such as elderly subjects.

##### Hyaluronic Acid (HA)

A natural substance found in the synovial fluid surrounding joints, HA is a polysaccharide from the glycosaminoglycan family that smoothly reduces attrition during movement, cushioning the shock between loads. Subjects with OA present lower HA levels. Therefore, exogenous HA infiltrations can help restore the elastic and viscous properties of the synovial fluid, minimizing pain and improving function. 12 Hyaluronic acid also increases shock absorption, provides synovial fluid lubrication, and acts on joint nociceptive response with reflex quadriceps muscle inhibition. The anti-inflammatory, analgesic, and chondroprotective action of HA results from the modulation of intra- and extracellular inflammation cascades. In addition to being safe and effective in treating pain due to patellar chondropathy, it improves mobility and slows down the degenerative process. 8

Russu et al. 41 investigated the treatment of knee OA with HA derivative infiltrations and observed satisfactory outcomes six months after the procedure, showing therapeutic efficiency in treating moderate to severe knee OA. In contrast, HA application alone yielded less significant outcomes concerning pain in patients under treatment when compared to HA combined with platelet-rich plasma (PRP). 42

However, some authors mention the occurrence of allergic side effects related to the product's origin. Pain and swelling after injection may result from the high molecular weight and different pharmaceutical formulations of HA. The other major challenge of HA treatment is the need for multiple injections to obtain the desired efficacy. As such, multiple injections could result in more cost, pain, and potential infection. Therefore, updates and innovations in single-dose high molecular weight medications have gained prominence in the therapeutic arsenal, with good outcomes in the joint functional status.

#### Indications

Infiltration of HA is an adjuvant therapy for OA treatment, mainly regarding symptoms and function, but its evidence still lacks scientific strength.

##### Platelet-Rich Plasma (PRP)

A blood-derived product with a high platelet concentration in a small plasma volume, PRP plays a role in the release of several growth factors (insulin-like growth factor 1 [IGF-1], platelet-derived growth factor [PDGF], epidermal growth factor [EGF], vascular epidermal growth factor [VEGF], and transforming growth factor-β [TGF-β]). All of these molecules act in the initial phase of healing and tissue regeneration. The type of growth factors in PRP and their levels vary in each subject. Moreover, its mechanism is not fully understood, and it is difficult to determine the effects of each type of factor. 43 The use of PRP in studies has already demonstrated considerable and complex biological activities, such as cell proliferation, anti-apoptotic activity, cartilage regeneration, collagen synthesis, and angiogenesis maximization. 43

Saita et al. 44 studied 517 patients receiving 3 PRP injections and analyzed the outcomes per the severity of knee OA. The authors state that the therapy is approximately 60% effective depending on the severity of the disease. Furthermore, this efficacy is independent of age, gender, body weight, or platelet count. 44 45 Knop et al., 46 based on RCTs, reported that PRP significantly improves joint pain and function when compared with HA, including sustained response for up to two years in milder knee OA cases.

Furthermore, it is worth highlighting that the Brazilian Federal Council of Medicine (Conselho Federal de Medicinas, CFM, in Portuguese), in its Resolution no. 2,128/2015, considers PRP an experimental procedure and only allows its use in clinical trials within the protocols of the Brazilian Research Ethics Committee/Research Ethics National Committee (Comitês de Ética em Pesquisa/Comissão Nacional de Ética em Pesquisa CEP/CONEP, in Portuguese) system. Likewise, the Brazilian National Health Regulatory Agency (Agência Nacional de Vigilância Sanitária, ANVISA, in Portuguese), in Technical Note no. 12/2015, ratified the experimental use of PRP as a product with no proven therapeutic utility, requiring more scientific evidence for clinical use.

#### Indications

Platelet-rich plasma is an adjuvant therapy for OA treatment, mainly concerning pain; it significantly improves pain and joint function compared to HA, including sustained response for up to two years in milder cases of knee OA.

##### Stem Cells

Stem-cell therapy emerges as a milestone in regenerative medicine for knee OA treatment, and its potential is deemed high. 47 Mesenchymal stromal/stem cells (MSCs), such as bone marrow, adipose tissue, and synovium stem cells, aid in damaged cartilage regeneration and are reportedly safe. 48

In a trial, 49 nine patients diagnosed with knee OA received a single injection of adipose-derived mesenchymal stem cells (AD-MSCs) at a concentration of 0.5–1.0 × 10 ^7^ cells. The follow-up period was of 18 months. The results showed significant improvement in all aspects evaluated in the initial six months, being sustained up to the end of the treatment. 49 An RCT by Chen et al. 50 assessed the safety and efficacy of allogeneic adipose-derived stem cells (ADSCs; ELIXCYTE, UnicoCell Biomed Co., Ltd., Taipei, Taiwan) infiltration for knee OA treatment. Patients either received one dose of HA or three doses of ELIXCYTE. ELIXCYTE was more effective than HA, providing an early pain reduction and functional improvements. 50

Although studies have shown that MSCs yield promising results in improving patients' clinical and structural conditions, there is still limited evidence, a lack of procedural standardization, and no long-term advantages.

#### Indications

Mesenchymal stem cells are adjuvant therapy for OA treatment, mainly for pain.

##### Prolotherapy with dextrose

The fundamental principle of dextrose prolotherapy is the injection of small volumes of an irritating solution into areas of painful attachments (ligaments, tendons, and adjacent joint spaces) at several treatment sessions. The mechanism of action remains unclear. However, hypotheses suggest that prolotherapy stimulates local healing of chronically-damaged extra- and intra-articular tissue, but with no definitive evidence. The literature 51 reports that prolotherapy injections may be appropriate to treat the multifactorial cause of OA-related knee pain.

#### Indications

Dextrose prolotherapy is an adjuvant method for OA treatment, mainly concerning pain, but there is no current evidence about it.

##### Genicular Nerve Block

In the literature survey, we retrieved seven randomized studies and one systematic review.

Genicular nerve block (GNB) is a therapeutic alternative aimed at blocking the three sensory nerves of the knee: the lateral and medial superior genicular nerve and the medial inferior genicular nerve. The block hinders the transmission of pain to the central nervous system and results in functional improvement. 52 Ultrasound can guide GNB, enabling direct visualization of the structures and real-time control of the anesthetic application. 53 Thus, the block is performed more efficiently, generating lower latency, dependence on anatomical information, and lower use of the anesthetic solution, in addition to providing greater safety. 54

A study 54 analyzed four elderly patients with advanced knee OA, presenting limited movement and chronic pain, who underwent ultrasound-guided GNB. The outcomes were satisfactory, with a significant improvement in pain and functional capacity and no complications. 54 A twelve-week analysis 55 compared the efficiency of IA block therapy and GNB. Both techniques resulted in satisfactory outcomes, minimizing pain, improving nighttime sleep quality, and facilitating the performance of daily activities. However, the study reports that GNB represents a safer, minimally-invasive, and highly-efficient alternative. 55

#### Indications

There is reasonable evidence to at least target the superior medial genicular nerve, the inferior medial genicular nerve, and the inferior medial genicular nerve using local anesthetics, corticosteroids, or alcohol to reduce pain and improve knee function in patients with chronic knee OA under ultrasound guidance. The procedure is safe, but more research is needed to determine the ideal interventional approach.

##### Acupuncture

Acupuncture is a well-known technique to treat several conditions by inserting fine needles in specific anatomical points. 56 Studies on acupuncture to minimize knee OA pain and provide functional rehabilitation have been increasing, demonstrating that this therapy acts to mitigate symptoms due to the activation of various chemical elements that present bioactivity through peripheral, spinal, and supraspinal mechanisms. 57

Teixeira et al. 6 evaluated the treatment of two patients with knee OA through one weekly, thirty-minute acupuncture session (for six weeks) at four local points. The researchers 6 reported pain minimization and increased range and mobility of the knee joint flexion, demonstrating that the technique is efficient as an alternative or complementary treatment for OA, even in a short period. In general, acupuncture can improve the subjective perception of the quality of life of patients with OA, enabling a better performance of daily activities. Nevertheless, one must consider factors such as the number and duration of acupuncture sessions, the patient's health status, and the time of prevalence of the disease.

#### Indications

Acupuncture plays a role in OA treatment, especially concerning pain.

##### Physical Exercises

Clinical guidelines for knee OA management suggest that patients should receive a core set of non-pharmacological interventions, including education, weight loss, and low-impact exercise (strengthening, cardiovascular exercise, and/or mind-body exercises, such as yoga or tai chi). 58 A recent systematic review 59 evaluated different RCTs on the effectiveness of land-based physical activities for patients with knee OA. In these studies, patients with this joint condition either underwent exercises or did not perform exercises or receive any treatment. The authors 59 found that the group performing land-based physical activities consistently presented reduced pain and improved their physical shape and quality of life.

#### Indications

Current research 60 indicates that aerobic activity for 150 minutes a week at moderate intensity, moderate/vigorous physical activity, or muscle strengthening exercises 2 days a week bring many benefits to subjects with preexisting knee OA.

##### Miscellaneous Interventions

Another therapeutic resource is the lateral wedge insole. Recent research 61 concluded that a customized external orthosis (fixed below the sole) resulted in a higher improvement in pain and functional status than a control orthosis. Likewise, varus or valgus correction orthoses (altering biomechanics) and shifting the load to the least compromised compartment are alternatives to gain function and improve pain.

## Final Considerations

Knee OA is a highly prevalent and disabling disease. In the past few years, we gained significant information about the cause and pathogenesis of OA, leading to a new era in OA therapy. However, management should not be generalized and requires adaptation according to each subject, enabling the establishment of the ideal intervention for each case.

Changing lifestyles, practicing physical exercise, and acupuncture, among others, are minimally-invasive methods for initial treatment, leaving surgical procedures as alternatives for cases in which these methods do not provide satisfactory responses. Therefore, the search for disease-modifying drugs for OA treatment has become a priority in orthopedics. Hyaluronic acid is safe and effective in treating OA-related pain in the knee and other joints, confirming that it may have some disease-modifying properties.

In conclusion, further studies on this topic are required to achieve better scientific knowledge and then understand and fill the remaining gaps.

## References

[1] Tan B Y Thach T Munro Y L Complex lifestyle and psychological intervention in knee osteoarthritis: Scoping review of randomized controlled trials Int J Environ Res Public Health 2021 18 23 12757 34886480 10.3390/ijerph182312757 PMC8657138

[2] Hunter D J Bierma-Zeinstra S Osteoarthritis Lancet 2019 393(10182):1745 1759 31034380 10.1016/S0140-6736(19)30417-9

[3] Salehi-abari I 2016 ACR Revised criteria for early diagnosis of knee osteoarthritis Autoimmune Dis Ther Approaches 2016 3 01 1 5

[4] Mobasheri A Batt M An update on the pathophysiology of osteoarthritis Ann Phys Rehabil Med 2016 59(5-6)333 339 27546496 10.1016/j.rehab.2016.07.004

[5] Mora J C Przkora R Cruz-Almeida Y Knee osteoarthritis: pathophysiology and current treatment modalities J Pain Res 2018 11 2189 2196 30323653 10.2147/JPR.S154002 PMC6179584

[6] Teixeira J Santos M J Matos L C Machado J P Evaluation of the effectiveness of acupuncture in the treatment of knee osteoarthritis: a case study Medicines (Basel) 2018 5 01 18 29401732 10.3390/medicines5010018 PMC5874583

[7] Tu J F Yang J W Shi G X Efficacy of intensive acupuncture versus sham acupuncture in knee osteoarthritis: a randomized controlled trial Arthritis Rheumatol 2021 73 03 448 458 33174383 10.1002/art.41584

[8] da Costa S R da Mota E Albuquerque R F Helito C P Camanho G L The role of viscosupplementation in patellar chondropathy Ther Adv Musculoskelet Dis 2021 13 X211015005 10.1177/1759720X211015005 PMC8127754 34035839

[9] Hart J M Kuenze C Norte G Prospective, randomized, double-blind evaluation of the efficacy of a single-dose hyaluronic acid for the treatment of patellofemoral chondromalacia Orthop J Sports Med 2019 7 06 2.325967119854192E15 10.1177/2325967119854192 PMC6593928 31263727

[10] Acharya K Si V Madi S Improvement in condition specific and generic quality of life outcomes in patients with knee osteoarthritis following single intraarticular viscosupplementation injection J Clin Orthop Trauma 2022 27 101828 35310785 10.1016/j.jcot.2022.101828 PMC8924687

[11] Silverwood V Blagojevic-Bucknall M Jinks C Jordan J L Protheroe J Jordan K P Current evidence on risk factors for knee osteoarthritis in older adults: a systematic review and meta-analysis Osteoarthritis Cartilage 2015 23 04 507 515 25447976 10.1016/j.joca.2014.11.019

[12] Koiri S P Yang Y Kui H Hyaluronic acid in the treatment of knee osteoarthritis. reviewYangtze Med 2018 2 62 72

[13] Heidari B Knee osteoarthritis prevalence, risk factors, pathogenesis and features: Part I Caspian J Intern Med 2011 2 02 205 212 24024017 PMC3766936

[14] de Rezende M U Gobbi R G Drug therapy in knee osteoarthrosis Rev Bras Ortop 2015 44 01 14 19 26998447 10.1016/S2255-4971(15)30043-4 PMC4783603

[15] Primorac D Molnar V Rod E Knee osteoarthritis: a review of pathogenesis and state-of-art non-operative therapeutic considerations Genes (Basel) 2020 11 08 1 35 10.3390/genes11080854 PMC7464436 32722615

[16] Boegård T Jonsson K [Hip and knee osteoarthritis. Conventional X-ray best and cheapest diagnostic method] Lakartidningen 2002 99 44 4358 4360 12469580

[17] Cho H Stuart J M Magid R Theranostic immunoliposomes for osteoarthritis. Nanomedicine Nanotechnology Biol Med (Aligarh) 2014 10 619 627 10.1016/j.nano.2013.09.004 PMC4962614 24096032

[18] Pires D Astur D Cohen M Classificações e fluxogramas em cirurgia do joelhoManole. Baueri - São Paulo: E-book;2021

[19] Kellgren J H Lawrence J S Radiological assessment of osteo-arthrosis Ann Rheum Dis 1957 16 04 494 502 13498604 10.1136/ard.16.4.494 PMC1006995

[20] Finan P H Buenaver L F Bounds S C Discordance between pain and radiographic severity in knee osteoarthritis: findings from quantitative sensory testing of central sensitization Arthritis Rheum 2013 65 02 363 372 22961435 10.1002/art.34646 PMC3863776

[21] OA Biomarkers Consortium Kraus V B Collins J E Hargrove D Predictive validity of biochemical biomarkers in knee osteoarthritis: data from the FNIH OA Biomarkers Consortium Ann Rheum Dis 2017 76 01 186 195 27296323 10.1136/annrheumdis-2016-209252 PMC5851287

[22] Tanishi N Yamagiwa H Hayami T Relationship between radiological knee osteoarthritis and biochemical markers of cartilage and bone degradation (urine CTX-II and NTX-I): the Matsudai Knee Osteoarthritis Survey J Bone Miner Metab 2009 27 05 605 612 19381754 10.1007/s00774-009-0077-3

[23] Hao H Q Zhang J F He Q Q Wang Z Cartilage oligomeric matrix protein, C-terminal cross-linking telopeptide of type II collagen, and matrix metalloproteinase-3 as biomarkers for knee and hip osteoarthritis (OA) diagnosis: a systematic review and meta-analysis Osteoarthritis Cartilage 2019 27 05 726 736 30391538 10.1016/j.joca.2018.10.009

[24] Felson D T Arthroscopy as a treatment for knee osteoarthritis Best Pract Res Clin Rheumatol 2010 24 01 47 50 20129199 10.1016/j.berh.2009.08.002 PMC2818323

[25] Losina E Weinstein A M Reichmann W M Lifetime risk and age at diagnosis of symptomatic knee osteoarthritis in the US Arthritis Care Res (Hoboken) 2013 65 05 703 711 23203864 10.1002/acr.21898 PMC3886119

[26] Su X Li C Liao W Comparison of arthroscopic and conservative treatments for knee osteoarthritis: a 5-year retrospective comparative study Arthroscopy 2018 34 03 652 659 29229416 10.1016/j.arthro.2017.09.023

[27] Zhao J Arthroscopic arthroplasty for knee osteoarthritis: denervation of subchondral bone and comprehensive synovectomy Arthrosc Tech 2021 10 12 e2651 e2657 35004145 10.1016/j.eats.2021.08.008 PMC8719106

[28] Giuffrida A Di Bari A Falzone E Conservative vs. surgical approach for degenerative meniscal injuries: a systematic review of clinical evidence Eur Rev Med Pharmacol Sci 2020 24 06 2874 2885 32271405 10.26355/eurrev_202003_20651

[29] Navarro R A Adams A L Lin C C Does Knee Arthroscopy for Treatment of Meniscal Damage with Osteoarthritis Delay Knee Replacement Compared to Physical Therapy Alone? Clin Orthop Surg 2020 12 03 304 311 32904116 10.4055/cios19114 PMC7449858

[30] Kang K S Tien T N Lee M C Lee K Y Kim B Lim D Suitability of Metal Block Augmentation for Large Uncontained Bone Defect in Revision Total Knee Arthroplasty (TKA) J Clin Med 2019 8 03 1 14 10.3390/jcm8030384 PMC6462980 30893934

[31] Jang S Lee K Ju J H Recent Updates of Diagnosis, Pathophysiology, and Treatment on Osteoarthritis of the Knee Int J Mol Sci 2021 22 05 2619 33807695 10.3390/ijms22052619 PMC7961389

[32] Peng H Ou A Huang X Osteotomy around the knee: the surgical treatment of osteoarthritis Orthop Surg 2021 13 05 1465 1473 34110088 10.1111/os.13021 PMC8313165

[33] Brouwer R W Huizinga M R Duivenvoorden T Osteotomy for treating knee osteoarthritis Cochrane Database Syst Rev 2014 2014 12 CD004019 25503775 10.1002/14651858.CD004019.pub4 PMC7173694

[34] He M Zhong X Li Z Shen K Zeng W Progress in the treatment of knee osteoarthritis with high tibial osteotomy: a systematic review Syst Rev 2021 10 01 56 33583421 10.1186/s13643-021-01601-z PMC7883424

[35] Paley D Tetsworth K Mechanical axis deviation of the lower limbs. Preoperative planning of uniapical angular deformities of the tibia or femur Clin Orthop Relat Res 1992 280 48 64 1611764

[36] Khakha R S Bin Abd Razak H R Kley K van Heerwaarden R Wilson A J Role of high tibial osteotomy in medial compartment osteoarthritis of the knee: Indications, surgical technique and outcomes J Clin Orthop Trauma 2021 23 101618 35070682 10.1016/j.jcot.2021.101618 PMC8758909

[37] Valeiy Evgenievich B Sergey Anatolievich M Liudmila Ivanovna A High tibial osteotomy in patients with stages 2 and 3 of knee osteoarthritis. Short-term result and factors affecting the outcome MOJ Orthop Rheumatol 2018 10 02 122 128

[38] Kan H S Chan P K Chiu K Y Non-surgical treatment of knee osteoarthritis Hong Kong Med J 2019 25 02 127 133 30919810 10.12809/hkmj187600

[39] Nguyen C Rannou F The safety of intra-articular injections for the treatment of knee osteoarthritis: a critical narrative review Expert Opin Drug Saf 2017 16 08 897 902 28627937 10.1080/14740338.2017.1344211

[40] Jones I A Togashi R Wilson M L Heckmann N Vangsness C T Jr Intra-articular treatment options for knee osteoarthritis Nat Rev Rheumatol 2019 15 02 77 90 30498258 10.1038/s41584-018-0123-4 PMC6390843

[41] Russu O M Pop T S Feier A M Treatment efficacy with a novel hyaluronic acid-based hydrogel for osteoarthritis of the knee J Pers Med 2021 11 04 303 33920879 10.3390/jpm11040303 PMC8071312

[42] Karasavvidis T Totlis T Gilat R Cole B J Platelet-rich plasma combined with hyaluronic acid improves pain and function compared with hyaluronic acid alone in knee osteoarthritis: a systematic review and meta-analysis Arthroscopy 2021 37 04 1277 12870 33278533 10.1016/j.arthro.2020.11.052

[43] Pintan G F de Oliveira A S Jr Lenza M Antonioli E Ferretti M Update on biological therapies for knee injuries: osteoarthritis Curr Rev Musculoskelet Med 2014 7 03 263 269 24986668 10.1007/s12178-014-9229-8 PMC4596155

[44] Saita Y Kobayashi Y Nishio H Predictors of effectiveness of platelet-rich plasma therapy for knee osteoarthritis: A retrospective cohort study J Clin Med 2021 10 19 4514 34640529 10.3390/jcm10194514 PMC8509123

[45] Bansal H Leon J Pont J L Platelet-rich plasma (PRP) in osteoarthritis (OA) knee: Correct dose critical for long term clinical efficacy Sci Rep 2021 11 01 3971 33597586 10.1038/s41598-021-83025-2 PMC7889864

[46] Knop E de Paula L E Fuller R Plasma rico em plaquetas no tratamento da osteoartrite Rev Bras Reumatol 2016 56 02 152 164 10.1016/j.rbre.2015.07.002 27267529

[47] Xiang X N Zhu S Y He H C Yu X Xu Y He C Q Mesenchymal stromal cell-based therapy for cartilage regeneration in knee osteoarthritis Stem Cell Res Ther 2022 13 01 14 35012666 10.1186/s13287-021-02689-9 PMC8751117

[48] Shariatzadeh M Song J Wilson S L The efficacy of different sources of mesenchymal stem cells for the treatment of knee osteoarthritis Cell Tissue Res 2019 378 03 399 410 31309317 10.1007/s00441-019-03069-9

[49] Spasovski D Spasovski V Baščarević Z Intra-articular injection of autologous adipose-derived mesenchymal stem cells in the treatment of knee osteoarthritis J Gene Med 2018 20 01 e-3002 10.1002/jgm.3002 29243283

[50] Chen C F Hu C C Wu C TE Treatment of knee osteoarthritis with intra-articular injection of allogeneic adipose-derived stem cells (ADSCs) ELIXCYTE®: a phase I/II, randomized, active-control, single-blind, multiple-center clinical trial Stem Cell Res Ther 2021 12 01 562 34717765 10.1186/s13287-021-02631-z PMC8557559

[51] Arias-Vázquez P I Tovilla-Zárate C A Legorreta-Ramírez B G Prolotherapy for knee osteoarthritis using hypertonic dextrose vs other interventional treatments: systematic review of clinical trials Adv Rheumatol 2019 59 01 39 31426856 10.1186/s42358-019-0083-7

[52] Lebleu J Fonkoue L Bandolo E Lower limb kinematics improvement after genicular nerve blockade in patients with knee osteoarthritis: a milestone study using inertial sensors BMC Musculoskelet Disord 2020 21 01 822 33287783 10.1186/s12891-020-03836-8 PMC7722305

[53] Pavão D M Rocha Faria J L Mandarino M Pulsed radiofrequency rhizotomy of the genicular nerves of the knee guided by radioscopy and ultrasonography: step-by-step technique Arthrosc Tech 2022 11 03 e391 e396 35256981 10.1016/j.eats.2021.11.006 PMC8897599

[54] Rodrigues T A de Oliveira E JSG Garcia J BS Pain management in patients with knee osteoarthritis by ultrasound-guided genicular nerve block. Case reports Br J Pain 2020 3 03 288 291

[55] Lauretti G R Santos D LR Oliveira C S Trintade C The Antinociceptive Effect of Genicular Nerves Block Compared to Intra-Articular Dexamethasone in Grade III or IV Knee Osteoarthritis J Biomed Sci Eng 2019 12 10 451 457

[56] Liu C Y Tu J F Lee M S Is acupuncture effective for knee osteoarthritis? A protocol for a systematic review and meta-analysis BMJ Open 2022 12 01 e052270 10.1136/bmjopen-2021-052270 PMC8753400 35017242

[57] Li J Li Y X Luo L J The effectiveness and safety of acupuncture for knee osteoarthritis: An overview of systematic reviews Medicine (Baltimore) 2019 98 28 e16301 31305415 10.1097/MD.0000000000016301 PMC6641846

[58] Bannuru R R Osani M C Vaysbrot E E OARSI guidelines for the non-surgical management of knee, hip, and polyarticular osteoarthritis Osteoarthritis Cartilage 2019 27 11 1578 1589 31278997 10.1016/j.joca.2019.06.011

[59] Fransen M McConnell S Harmer A R Van der Esch M Simic M Bennell K L Exercise for osteoarthritis of the knee: a Cochrane systematic review Br J Sports Med 2015 49 24 1554 1557 26405113 10.1136/bjsports-2015-095424

[60] Dantas L O Salvini T F McAlindon T E Knee osteoarthritis: key treatments and implications for physical therapy Braz J Phys Ther 2021 25 02 135 146 33262080 10.1016/j.bjpt.2020.08.004 PMC7990728

[61] Reichenbach S Felson D T Hincapié C A Effect of Biomechanical Footwear on Knee Pain in People With Knee Osteoarthritis: The BIOTOK Randomized Clinical Trial JAMA 2020 323 18 1802 1812 32396180 10.1001/jama.2020.3565 PMC7218497

