# Supplementary material for: Updates in the Treatment of Knee Osteoarthritis
Source: Rev Bras Ortop (Sao Paulo). 2024 Jun 22;59(3):e337–48. doi: 10.1055/s-0044-1786351 (PMC11193585; doi:10.1055/s-0044-1786351)
Supplement: Related texts

# Research article: Atualizações no tratamento da osteoartrite de joelho

**Keywords:** artroscopia, biomarcadores, cartilagem articular, exercício físico, joelho, infiltrações intra-articulares, osteoartrite

## Abstract

A osteoartrite (OA) do joelho é uma doença inflamatória e degenerativa que ocasiona a destruição da cartilagem articular e leva à perda de funções. Sua prevalência vem crescendo consideravelmente devido ao aumento da expectativa de vida e da obesidade, e o diagnóstico pode ser feito por meio de avaliação e exames médicos, e é confirmado em imagens radiográficas complementares. Uma condição multifatorial, a OA do joelho pode ser influenciada por diversos aspectos locais, sistêmicos e externos; além disso, a sua evolução e as respostas aos tratamentos dependem muito das características de cada indivíduo. Inicialmente, recomenda-se proceder a um tratamento medicamentoso e a terapias alternativas que melhorem a qualidade de vida do paciente; mas, a partir do momento em que se verifica que tais terapias não estão proporcionando resultados satisfatórios, um tratamento cirúrgico deve ser considerado. Entre os tratamentos cirúrgicos, as artroscopias, as osteotomias e as artroplastias parciais e totais são destacadas; os métodos não cirúrgicos incluem o uso de medicamentos e de terapias alternativas, como infiltrações, acupuntura e prática de exercícios físicos. Vale ressaltar ainda que a utilização de biomarcadores pode ser uma importante estratégia para detectar precocemente a doença, avaliar sua atividade, prever um prognóstico e monitorar uma melhor resposta à terapia; porém, esse tema ainda deve ser foco de mais estudos para que os seus resultados sejam comprovados.

## Introdução

A osteoartrite (OA) é uma doença responsável pela degradação da cartilagem, remodelação óssea, formação de osteófitos e inflamação sinovial; causa dores, características de rigidez, edema e perda da funcional articular, e é uma das principais causas de inabilidade entre as faixas etárias adulta e idosa. 1 2 Em decorrência do envelhecimento da população e a progressiva prevalência da obesidade, esta doença vem apresentando maior incidência nos dias atuais. 2

A OA é considerada a doença mais comum do joelho que, quando instalada, pode ocasionar derrame articular de repetição e limitação funcional. 3 Nas últimas décadas, verificou-se lentidão e constância na compreensão da fisiopatologia da OA do joelho; contudo, lacunas referentes à sua patogênese causam impactos negativos no desenvolvimento e na inovação de medicamentos. Devido à falta de novos medicamentos e drogas modificadoras para tratar OA do joelho, torna-se evidente a necessidade de alteração de estilo de vida, o quem pode retardar a necessidade de intervenção cirúrgica. 4 Além disso, a atividade física de baixo impacto recebe destaque pelas sociedades médicas, ao passo que as intervenções cirúrgicas podem mostrar resultados conflitantes. 5 6 7

Adicionalmente, as terapias biológicas vêm sendo cada vez mais procuradas como alternativas para o tratamento da OA, com destaque para as infiltrações intra-articulares (IAs). O ácido hialurônico (AH) é amplamente utilizado como tratamento conservador seguro para OA no joelho e outras articulações, 8 com o objetivo de proporcionar alívio da dor, estimular a produção de líquido sinovial e fornecer amortecimento e lubrificação às superfícies articulares. 9

Há uma crescente vertente científica que apoia uso das IAs com AH para OA de joelho em detrimento de placebo e de outros tipos de terapias. Acharya et al., 10 ao analisarem a eficácia da IA com AH em termos de dor, capacidade funcional e qualidade de vida em pacientes com OA do joelho precoce e primária, relataram efeitos benéficos de curto prazo (até um ano), e observaram uma tendência decrescente na intensidade da dor e uma progressiva melhora do funcionamento físico e da qualidade de vida relacionada à saúde do paciente, após uma única aplicação.

Uma condição multifatorial, a OA do joelho pode ser influenciada por diversos aspectos locais, sistêmicos e externos; além disso, a sua evolução e as respostas aos tratamentos dependem muito das características de cada indivíduo. Visando um melhor entendimento sobre essa patologia, o objetivo deste estudo foi reunir informações sobre a fisiopatologia e o diagnóstico clínico, bem como trazer atualizações sobre o seu tratamento.

## Fisiopatologia da osteoatrite de Joelho

A OA é uma doença decorrente de processos biológicos complexos, que incluem cartilagem, osso, ligamentos, sinóvia, menisco, gordura periarticular e músculos. Comumente, a OA é caracterizada pelo estreitamento do espaço articular (EEA) devido à perda de cartilagem articular, degeneração meniscal e alterações ósseas, incluindo esclerose do osso subcondral e osteófitos. 11 12

O desenvolvimento da doença pode ser influenciado pelo ambiente biomecânico, em que o alinhamento em varo desloca a carga medialmente, o que potencializa o risco de OA do joelho do compartimento medial, ao passo que o alinhamento em valgo desloca a carga lateralmente e desencadeia a OA do compartimento lateral. Adicionalmente, a ocorrência prévia de trauma no joelho aumenta o risco de OA em 3,86 vezes. 13

Geralmente, uma sobrecarga mecânica é responsável por iniciar o processo de danificação da cartilagem, que posteriormente evolui para uma inflamação, o que causa a degradação das articulações. O processo de inflamação tem como seus percursores primários a interleucina-1 (IL-1) e o fator de necrose tumoral (TNF), responsáveis pela indução da maximização da expressão de metaloproteinases e óxido nítrico, que são os principais agentes catabólicos fabricados pelos condrócitos como resposta à lesão. 14 Portanto, pode-se dizer que a OA é uma doença inflamatória de toda a articulação sinovial, que compreende a degeneração mecânica e estrutural da cartilagem articular e desencadeia alterações funcionais de toda a articulação. 4

Essa doença se desenvolve de forma lenta, e a dor causada por ela pode ser progressiva, 12 o que a torna uma das enfermidades que mais causam dor e inabilidade, pois afeta diretamente a qualidade de vida do indivíduo. 2 A sua ocorrência pode se dever a diversos fatores, como faixa etária, distúrbios de peso corporal, sobrecarga articular do joelho, hiperflexão prolongada, desvios de eixo, entre outros. 3

Primorac et al. 15 mencionam que a prevalência de OA do joelho é mais alta entre adultos com idade de 60 anos ou mais, e representa uma das principais causas de incapacidade entre idosos, sendo o risco maior em pessoas obesas (índice de massa corporal [IMC] ≥ 30 kg/m ^2^ ) do que em pessoas não obesas (19,7% *versus* 10,9%).

## Diagnóstico Clínico e Sistemas de Classificação

O diagnóstico inicialmente é clinico, realizado com base nos sintomas de dor, rigidez e limitações funcionais, bem como em um minucioso exame físico, que envolve a observação de crepitação, dor e restrição na mobilidade, sensibilidade articular e aumento ósseo. 2 A observação de radiografias com carga dos joelhos é indicada para ajudar na confirmação diagnóstica, assim como na classificação do grau da OA, o que possibilita a verificação do dano estrutural e melhora a especificidade quando os osteófitos ou o EEA estão presentes. 16

A utilização de ressonância magnética (RM) também possibilita a visualização dos efeitos causados pela OA do joelho na cartilagem, no menisco, na membrana sinovial e no osso subcondral. Além disso, uma ferramenta desenvolvida por Cho et al. 17 direciona imunolipossomas de infravermelho a anticorpos de colágeno de tipo II para o diagnóstico e o tratamento precoces de OA.

As classificações desempenham um papel fundamental na cirurgia do joelho, pois uma estimativa prognóstica pode ser orientada por sistemas de classificação eficazes. A recente publicação de Pires et al. 18 , “Classificações e fluxogramas em cirurgia do joelho”, é uma obra de referência brasileira que reúne as diversas classificações, fluxogramas e questionários da área, e auxilia no diagnóstico e na tomada de decisão por parte do especialista.

Segundo as classificações para lesões degenerativas, 18 de maneira geral, o método mais comum para a definição radiográfica é o sistema de classificação Kellgren-Lawrence. A pontuação geral da articulação classifica a OA com os seguintes graus: grau 0–nenhum achado patológico; grau 1–EEA duvidoso e possíveis osteófitos na borda; grau 2–possível EEA e osteófitos definidos; grau 3–EEA definido, múltiplos osteófitos, alguma esclerose subcondral, e possível deformidade do contorno ósseo; e grau 4–EEA notável, esclerose subcondral severa, deformidade do contorno ósseo definida e presença de osteófitos grandes. 19

## Exames clínico e de imagem

Cerca de 40% da população com mais de 65 anos apresenta OA do joelho sintomática. A detecção e intervenção precoces são de suma importância para diminuir a morbidade e a incapacidade, para proporcionar uma vida independente melhor. A radiografia é a primeira investigação de escolha para pacientes com OA que apresentam dor no joelho. Estudos baseados em comunidades 19 mostraram que 40% a 80% dos indivíduos com OA com alteração radiográfica do joelho são sintomáticos, e que achados radiográficos graves estão associados a relatos de dor de dor mais intensa. No entanto, há um alto grau de discordância entre os achados clínicos e radiográficos, e a classificação radiológica da OA é imprecisa nos estágios iniciais. O motivo da discordância entre dor e radiografia pode ser explicado pela origem multifatorial da dor. 19

A discordância entre os exames físico e de imagem, principalmente em relação aos sintomas, pode ser explicada pela propensão de alguns pacientes com OA a desenvolver circuitos nociceptivos centrais sensibilizados que aumentam a dor durante vários estados de agressão aos tecidos periféricos. 20 21 Essa anormalidade, conhecida como sensibilização central, é um processo nociceptivo mal-adaptativo que envolve alterações neuroplásticas complexas que amplificam a dor em vários níveis do neuroeixo. 20 Uma vez que a sensibilização central está correlacionada com a ativação de circuitos neurais que estão implicados na facilitação descendente da dor e é, portanto, um fator de risco para o desenvolvimento e manutenção da dor crônica, é importante identificar quais pacientes apresentam respostas anormais a estímulos dolorosos relevantes.

## Biomarcadores

Um biomarcador preciso e confiável tem que apresentar especifidade para uma determinada patologia e ter a capacidade de refletir a atividade real da doença, acompanhando as alterações alcançadas com a terapia e, ainda, ser capaz de prever o prognóstico. 20 Desta forma, a maioria dos marcadores bioquímicos de OA caracterizam a renovação da cartilagem. Os marcadores mais comumente investigados são: para a degradação da matriz extracelular ( *extracellular matrix* , ECM, em inglês) – fragmentos de C-telopeptídeo de colágeno tipo II urinário ( *urinary C-telopeptide fragments of type-II collagen* , uCTX-II, em inglês), Coll2–1, C2C, C2M, Coll2–1NO2, proteína oligomérica da matriz da cartilagem ( *cartilage oligomeric matrix protein* , COMP, em inglês), epítopos de agrecano (ARGS, TEGE, FFGV), epítopos de fibulina-3 (Fib3–1, Fib3–2, Fib3–3) etc.; e, para a síntese da ECM – PIIANP, PIIBNP, CPII, CS846, entre outros. 21 O uCTX-II é um dos marcadores bioquímicos de OA mais conhecidos, e apresenta um perfil preditivo superior quando comparado aos outros.

Segundo a literatura, várias condições podem afetar os níveis de biomarcadores. Algumas evidências sugerem que atividade física, níveis hormonais no corpo, medicações e o sexo do paciente podem ocasionar flutuações nos níveis de biomarcadores. Tanishi et al. 22 observaram que os níveis de uCTX-II diferiram significativamente entre os sexos e em mulheres na pré-menopausa e pós-menopausa.

Devido à sua capacidade de distinguir entre indivíduos saudáveis e pacientes com OA, outro potencial marcador bioquímico amplamente divulgado na literatura é o COMP. 23 Além disso, no caso do COMP, foram observadas possíveis capacidades prognósticas. Embora alguns estudos tenham relatado resultados conflitantes, uma metanálise 23 mostrou que esses marcadores bioquímicos (isto é, COMP e CTX-II) podem ser eficazes para o diagnóstico de OA, o prognóstico de progressão e a diferenciação entre grupos saudáveis e indivíduos afetados por OA.

O surgimento desses tipos de biomarcadores é relativamente recente, e ainda estão sendo estudadas as melhores estratégias para a sua aplicação, em termos de tecnologia e pesquisa médica, com o objetivo de desenvolver métodos de detecção que demonstrem confiabilidade.

## Tratamentos

O tratamento de primeira linha para a OA do joelho não envolve intervenções cirúrgicas, e pode ser manejado de forma multimodal. Porém, a partir do momento em que se identifica que tais terapias não foram satisfatórias, um tratamento cirúrgico deve ser considerado. Ainda que não exista cura para a OA do joelho, há várias opções de tratamento cirúrgico e não cirúrgico para auxiliar na administração da dor e na manutenção da saúde dos pacientes. 12

Na seção seguinte, serão discutidos os tratamentos mais difundidos conforme o seu nível de recomendação na atualidade.

## Materiais e Métodos

Estratégia de pesquisa

Foi realizado levantamento bibliográfico de estudos publicados até 20 de fevereiro de 2023 nos bancos de dados PubMed, MEDLINE e Embase. Foram utilizados os seguintes descritores: *Updates* ; *Knee Osteoarthrosis* ; *Treatment* . Os seguintes filtros foram utilizados para se chegar ao resultado final esperado: *Randomized Controlled Trial* ; *Randomized Clinical Trial* ; *Meta-Analysis* ; *Systematic Reviews* ; *Cohort* .

## Critérios de Elegibilidade e Seleção

Os estudos encontrados foram submetidos aos seguintes critérios de inclusão:

- Estudos relacionados ao tema proposto;
- Estudos com nível de evidência de I a III;
- Artigos em inglês, espanhol e português;
- Pesquisas realizadas em humanos; e
- Artigos disponíveis na versão completa.

E o critério de exclusão foi:

- Estudos de nível de evidência baixo, como relatos de caso simples.

## Resultados

## Identificação dos Estudos e Características

Segundo a estratégia de busca, foram encontrados 41 estudos com os descritores supracitados, os quais foram novamente avaliados segundo seu desenho e relevância, conforme os filtros do tipo de estudo e os critérios de inclusão. Ao final, restaram oito estudos que estavam disponíveis na versão completa e foram incluídos segundo a categoria e o tipo de tratamento.

## Tratamentos Cirúrgicos

## Artroscopia

A artroscopia é uma técnica realizada com o intuito de retirar os detritos e os cristais liberados na cavidade das articulações com OA, o que regulariza a superfície articular. 24 No levantamento da literatura, foram encontradas 26 revisões sistemáticas e metanálises que, somadas, descreveram resultados de 6.418 pacientes, com idade média de 47 ± 19 anos ( Figs. 1 e 2 ).

**Fig. 1:** Representação gráfica dos desfechos apresentados nos estudos que avaliaram o desempenho da artroscopia no tratamento de doenças degenerativas do joelho.

**Fig. 2:** Descrição temporal da literatura incluída por ano de publicação.

A utilização da cirurgia artroscópica para a doença degenerativa do joelho tem sido o foco de vários ensaios clínicos randomizados (ECRs) publicados recentemente, assim como de revisões sistemáticas de alta qualidade e metanálises. Apesar de estudos pioneiros terem demonstrado que a artroscopia minimamente invasiva apresenta resultados promissores, a força destes trabalhos não é suficiente para repercutir como recomendação. Atualmente, estudos clínicos que comparam o desbridamento articular artroscópico com cirurgia placebo ou tratamento conservador demonstraram que a artroscopia não é mais eficaz, e não tem grau de recomendação. 10 11 12 13 14 15 16 17 25

Uma recente revisão sistemática 11 publicada no *British Medical Journal* produziu uma forte recomendação contra o uso de artroscopia em pacientes com doença degenerativa do joelho, e enfatizou a importância de utilizar totalmente as opções apropriadas de tratamento conservador. Em particular, as recomendações direcionam os médicos a maximizar a terapia de exercícios, pois os resultados apresentados nos ECRs demonstram que os resultados das cirurgias artroscópicas não são superiores aos resultados encontrados em pacientes que seguiram protocolo de fisioterapia completo. 11 Outra revisão sistemática, de oito ECRs com avaliação da cirurgia artroscópica no cenário de lesões meniscais degenerativas, encontrou evidências moderadas que sugerem que não há nenhum benefício clínico do desbridamento meniscal artroscópico para lesões meniscais degenerativas em comparação com tratamentos não cirúrgicos ou simulados em pacientes de meia-idade com OA. 10 26 27 28

Neste contexto, Navarro et al., 29 em uma coorte prospectiva, tiveram como objetivo responder se a artroscopia de joelho para o tratamento de danos meniscais por OA atrasa a substituição do joelho em comparação com a fisioterapia isolada. Um dos principais achados foi que a incidência cumulativa de substituição do joelho foi modesta, mas significativamente maior para aqueles que receberam artroscopia do que para aqueles que foram submetidos apenas a fisioterapia (razão de risco: 1,30; intervalo de confiança de 95%: 1,17–1,44; *p* < 0,001). Os autores concluíram que, para pacientes com dano meniscal complicado por OA, aqueles que foram submetidos a artroscopia tiveram 30% mais chances de ter uma cirurgia de substituição parcial ou total do joelho em qualquer momento do acompanhamento do que aqueles que foram tratados apenas com fisioterapia.

## Indicações

Por todas estas razões, muitos pesquisadores limitam as indicações da artroscopia. A técnica ainda apresenta vantagens em estágios iniciais de OA, em que a limitação do movimento é mecânica, com a presença de corpos livres articulares, mas o seguimento do tratamento deve obrigatoriamente ser feito com fisioterapia. 26

Zhao et al. 27 explicam que, entre as várias opções cirúrgicas, a artroscopia não é vantajosa devido às evidências fracas e escassas que apoiam a sua aplicação. Da mesma forma, estudos recentes 28 sugerem que a artroscopia pode não ser benéfica para pacientes com rotura meniscal degenerativa crônica, e enfatizam a importância do seguimento conservador para o manejo da OA do joelho.

O benefício pequeno e irrelevante observado nas intervenções que incluem artroscopia para o joelho degenerativo é limitado no tempo e ausente um a dois anos após a cirurgia. A artroscopia do joelho está associada a malefícios. Tomados em conjunto, esses achados não apoiam a prática de cirurgia artroscópica para pacientes de meia-idade ou idosos com dor no joelho, com ou sem sinais de OA.

## Artroplastia

A artroplastia é uma opção cirúrgica para o tratamento de pacientes com quadros extremos de degeneração articular no joelho. A artroplastia do joelho pode ser total (ATJ) ou unicompartimental (AUJ), dependendo do grau de OA. 30

No levantamento da literatura, foram encontradas 22 revisões sistemáticas e metanálises que, somadas, descreveram resultados de 14.095 pacientes, com idade média de 58 ± 21 anos ( Figs. 3 e 4 ).

**Fig. 3:** Representação gráfica dos desfechos apresentados nos estudos que avaliaram o desempenho da artroplastia (total ou parcial) no tratamento de doenças degenerativas do joelho.

**Fig. 4:** Descrição temporal da literatura incluída por ano de publicação.

Se a OA for limitada a um compartimento, a AUJ ou a osteotomia de descarga podem ser consideradas. Eles são recomendadas em pacientes jovens e ativos devido aos riscos e à durabilidade limitada da ATJ. Já a ATJ é um método comum e seguro em pacientes idosos com OA de joelho avançada, sendo considerado o tratamento cirúrgico padrão-ouro para casos avançados. 30

Um ECR 31 de ATJ demonstrou que o tratamento cirúrgico após OA é superior ao tratamento não cirúrgico isolado no alívio da dor em pacientes com OA moderada a grave do joelho e na melhora da função e qualidade de vida após um ano. No entanto, melhoras clinicamente relevantes foram observadas em ambos os grupos, e os pacientes que receberam ATJ tiveram efeitos colaterais mais graves. Além disso, a ressecção do coxim adiposo interpatelar durante a ATJ é objeto de um debate contínuo, sem consenso claro. 31

## Indicações

A ATJ é indicada para pacientes com processo de degeneração articular avançada, e também é tida como vantajosa em idosos acima de 60 anos, sendo padrão-ouro nessas condições. A AUJ é recomendada em pacientes jovens e ativos devido aos riscos e à durabilidade limitada da ATJ.

## Osteotomia

A osteotomia tibial alta (OTA) vem sendo utilizada há mais de sessenta anos como exercício clínico, sendo uma técnica realizada em torno do joelho com o objetivo de manter a estruturação anatômica normal e alcançar uma melhor recuperação funcional da articulação do joelho, além de atenuar a maciez articular e abrandar o modo de degeneração da cartilagem, o que prevene ou adia a substituição das articulações. 32 Essa técnica é realizada por meio de uma osteotomia controlada com a transferência do eixo de suporte de peso do compartimento com degeneração para o compartimento que se encontra saudável, visando o realinhamento do membro inferior. 33 Consequentemente, esse procedimento apresenta pouca interferência nos tecidos moles, e geralmente não apresenta efeitos prejudiciais à estabilidade e à mobilidade da articulação do joelho. 34

No levantamento da literatura, foram encontradas 19 revisões sistemáticas e metanálises que, somadas, descreveram resultados de 8.412 pacientes, com idade média de 51 ± 7 anos ( Figs. 5 e 6 ).

**Fig. 5:** Representação gráfica dos desfechos apresentados nos estudos que avaliaram o desempenho da osteotomia no tratamento de doenças degenerativas do joelho.

**Fig. 6:** Descrição temporal da literatura incluída por ano de publicação.

A OTA compreende duas técnicas principais: a OTA em cunha de fechamento (OTACF) e OTA em cunha de abertura (OTACA). 34 Tais procedimentos podem ser realizados proximal ou distalmente à articulação do joelho: de forma geral, se a OA de joelho se dá no compartimento medial, a OTA é mais comumente feita pela operação na tíbia superior; se a OA se encontrar no compartimento lateral, a OTA é geralmente realizada no fêmur inferior. No entanto, é recomendável sempre mensurar os ângulos femoral lateral distal e o ângulo tibial proximal medial, visando otimizar o local correto da realização da osteotomia e se ela será de adição ou de subtração, seguindo os princípios descritos por Paley e Tetsworth. 35

A OTA vem sendo direcionada como procedimento para indivíduos jovens e ativos, geralmente na faixa etária menor do que 50 anos. Os resultados obtidos com a técnica podem ser influenciados por diversos fatores. 36 Valeiy Evgenievich et al. 37 demonstraram uma maior eficácia da OTA no segundo estágio da OA de joelho, quando comparada ao terceiro estágio, e observaram ainda que a faixa etária e o ângulo de correção não afetam o resultado, mas altos índices de massa corporal são associados a piores resultado, assim como à complicações. A sua imprecisão é designada de forma heterogênea na literatura, que compreende resultados funcionais mais baixos, progressão da OA e conversão para artroplastia.

Por fim, esta terapia é considerada tratamento autônomo, mas o processo de alinhamento dos membros relaciona-se intrinsicamente com as estruturas articulares e estabilizadoras do joelho. Assim, pode ser considerada em combinação com outras osteotomias e artroplastias, e, ainda, como estratégia de tratamento na realização de procedimentos para estabilizar o joelho e restaurar a cartilagem.

## Indicações

A OTA é muito indicada para pacientes jovens, principalmente em casos de OA com cartilagem parcialmente preservada. Para a correção da biomecânica do joelho, a OTA é mais eficaz para pacientes com OA de joelho no segundo estágio do que no terceiro estágio.

Já a osteotomia fibular proximal (OFP) é mais indicada para pacientes com muitas complicações e baixa tolerância cirúrgica, pois pode reduzir o tempo de operação, o sangramento intraoperatório, a internação e as complicações pós-operatórias, o que tem algumas vantagens.

## Tratamentos Não Cirúrgicos

## Terapias Farmacológicas

A terapia farmacológica para a OA é puramente sintomática; na maioria dos casos, garante com sucesso a manutenção da mobilidade do paciente, e pode incluir o uso de paracetamol, anti-inflamatórios não esteroidais (AINEs) tópicos ou orais, ou corticosteroides intra-articulares. Apesar de o paracetamol apresentar-se como um medicamento de considerável eficácia, segurança e custo, as diretrizes da Osteoarthritis Research Society International (OARSI) recomendam que seja administrado em doses e por durações conservadoras, pois há preocupação com o aumento do risco de distúrbios gastrointestinais e alterações hepáticas. 38

Em paralelo, os AINEs são o tratamento farmacológico de primeira linha para a OA, e têm sua evidência comprovada a partir de ensaios controlados por placebo. Os AINEs tópicos geralmente apresentam menos toxicidade gastrointestinal do que os orais. Contudo, para os pacientes suscetíveis ou que apresentem fatores de riscos aos efeitos adversos da medicação, alternativas aos AINEs devem ser discutidas. Vale destacar que o uso de medicamentos deve ser realizado como complementação, não como substituição para tratamentos que não utilizam medicamentos.

## Indicações

Para pacientes com OA sintomática.

## Infiltrações Intra-articulares

As terapias biológicas vêm sendo cada vez mais procuradas como alternativas para o tratamento da OA; assim, destacam-se as IAs, que são tratamentos aprovados pela Food and Drug Administration (FDA) dos Estados Unidos e pela Agência Europeia de Medicamentos (European Medicines Agency, EMA, em inglês). 39 As infiltrações IAs possibilitam a administração de uma dosagem concentrada de um medicamento, que é distribuido de forma local e por toda a articulação. Assim, o medicamento pode acessar locais inacessíveis com a ingestão de medicamentos orais. Além disso, as infiltrações IAs oferecem uma biodisponibilidade muito melhor e limitam a passagem sistêmica de medicamentos, reduzindo o risco de efeitos colaterais. 39 40

## Indicações

Indicadas como terapia adjuvante para o tratamento de OA, principalmente em pacientes que já fazem tratamento sistêmico crônico, como pacientes idosos.

## Ácido hialurônico (AH)

O AH é uma substância natural encontrada no fluido sinovial que envolve as articulações. Trata-se de polissacarídeo da família dos glicosaminoglicanos que permite a diminuição do atrito na realização do movimento, de forma suave, o que amortece o choque entre as cargas. Indivíduos com OA apresentam menores concentrações dessa substância; por isso, infiltrações de AH exógeno podem auxiliar na restauração das propriedades elásticas e viscosas do líquido sinovial, o que minimiza a dor e proporciona a melhora da função. 12 Essa substância ainda proporciona melhora das propriedades de absorção de choque, lubrificação do líquido sinovial, e da resposta nociceptiva articular, com a inibição muscular reflexa no quadríceps. As ações anti-inflamatória, analgésica e condroprotetora do AH relacionam-se à modulação da cascata de inflamação intra e extracelular. O AH também apresenta segurança e eficácia na terapia de dores relacionadas à condropatia patelar, ao atuar na melhora da mobilidade e na desaceleração do processo degenerativo. 8

Russu et al., 41 ao investigarem o tratamento de OA do joelho com infiltrações de derivados de AH, observaram resultados satisfatórios após seis meses do procedimento, o que mostra a eficácia terapêutica no tratamento da OA do joelho de moderada a grave. Por outro lado, a aplicação de AH isolado demonstrou resultados menos significativos em relação às dores de pacientes em tratamento de OA quando comparada à combinação de AH com plasma rico em plaquetas (PRP). 42

No entanto, alguns autores citam a ocorrência de efeitos colaterais alérgicos relacionados à origem desse produto. Dor e inchaço após a injeção podem estar relacionados ao alto peso molecular e às diferentes formulações farmacêuticas de AH. O outro grande desafio do tratamento com AH é a necessidade de múltiplas injeções para se obter a eficiência desejada. Dessa maneira, injeções múltiplas poderiam acarretar mais custo, dor e possibilidade de infecção. Neste sentido, atualizações e inovações nas medicações de alto peso molecular em dose única têm ganhado destaque no arsenal terapêutico, com bons resultados no quadro funcional articular.

## Indicações

Indicado como terapia adjuvante para o tratamento de OA, principalmente em relação aos sintoma e à função, mas as evidências dessa terapia ainda carecem de força científica.

## Plasma Rico em Plaquetas (PRP)

O PRP é um produto derivado do sangue que apresenta elevada concentração de plaquetas em um pequeno volume de plasma. Esse produto é responsável pela liberação de uma variedade de mediadores e fatores de crescimento, como o fator de crescimento semelhante à insulina tipo 1 ( *insulin-like growth factor 1* , IGF-1, em inglês), o derivado de plaquetas ( *platelet-derived growth fator* , PDGF, em inglês), o de crescimento epidérmico ( *epidermal growth fator* , EGF, em inglês), EGF vascular ( *vascular* EGF, VEGF, em inglês), e o de crescimento transformador-β ( *transforming growth factor-β* , TGF-β, em inglês), que atuam na fase inicial de cura e regeneração tecidual. O tipo e as concentrações do fator de crescimento presente no PRP são variáveis em cada indivíduo, e o seu mecanismo não é totalmente esclarecido; por isso, existe dificuldade na determinação dos efeitos de cada tipo de fator. 43 A utilização de PRP em estudos já demonstrou atividades biológicas consideráveis e complexas, como proliferação celular, atividade antiapoptótica, regeneração de cartilagem, síntese de colágeno, e maximização da angiogênese. 43

Saita et al. 44 realizaram uma pesquisa com 517 pacientes que foram submetidos a 3 injeções de PRP e analisaram os resultados de acordo com a gravidade da OA de joelho. Os autores afirmam que a terapia tem aproximadamente 60% de eficácia, e que depende da gravidade da doença. Além disso, essa eficácia é independente de faixa etária, gênero, peso corpóreo ou contagem de plaquetas. 44 45 Knop et al. 46 também verificaram que, com base em ECRs, o PRP produz uma melhora significativa na dor e na função articular quando comparado ao AH, que compreende a sustentação da resposta por até dois anos, evidenciada em casos mais leves de OA do joelho.

Além disso, é importante ressaltar que o Conselho Federal de Medicina (CFM), na sua Resolução n ^o^ 2.128/2015, considera o PRP um procedimento experimental, que só pode ser utilizado em experimentação clínica dentro dos protocolos do sistema Comitês de Ética em Pesquisa/Comissão Nacional de Ética em Pesquisa (CEP/CONEP). Da mesma forma, a Agência Nacional de Vigilância Sanitária (ANVISA), por meio da Nota Técnica n ^o^ 12/2015, ratificou o uso experimental do PRP por considerá-lo um produto sem utilidade terapêutica comprovada e que necessita de mais evidência científica para seu uso clínico.

## Indicações

Indicado com terapia adjuvante para o tratamento de OA, principalmente em relação à dor. Conforme já dito, o PRP produz uma melhora significativa na dor e na função articular quando comparado ao AH, que compreende a sustentação da resposta por até dois anos, evidenciada em casos mais leves de OA do joelho.

## Células-tronco

A terapia com células-tronco surge como um marco na medicina regenerativa para o tratamento de OA de joelho, e o seu emprego é considerado um dos tratamentos de maior potencialidade. 47 A utilização de células-tronco/estromais mesenquimais ( *mesenchymal stromal/stem cells* , MSCs, em inglês), como as da medula óssea, do tecido adiposo e da sinóvia, auxilia na regeneração da cartilagem danificada e é descrita como uma terapia segura. 48

Uma pesquisa 49 incluiu 9 pacientes diagnosticados com OA do joelho tratados com apenas uma injeção de células-tronco derivadas do tecido adiposo ( *adipose-derived mesenchymal stem cells* , AD-MSCs, em inglês) na concentração de 0,5–1,0 × 10 ^7^ células e acompanhados por 18 meses. Os resultados mostraram melhora significativa de todos os aspectos avaliados nos 6 meses iniciais, que se manteve até o final do tratamento. 49 A segurança e a eficácia da infiltração de células-tronco alogênicas derivadas do tecido adiposo ( *adipose-derived stem cells* , ADSCs, em inglês; ELIXCYTE, UnicoCell Biomed Co., Ltd., Taipei, Taiwan) para o tratamento de OA do joelho foi avaliado por Chen et al. 50 em um ECR. Os pacientes foram divididos em grupos que receberam ou uma dose de AH ou três doses de ELIXCYTE. Os autores verificaram maior eficiência da ELIXCYTE, a qual proporcionou uma redução precoce de dor e melhoras funcionais. 50

Embora estudos demonstrem que a terapia com MSCs apresenta resultados promissores na melhora do quadro clínico e estrutural dos pacientes, ainda existe limitação de evidências e falta de padronização de procedimentos, além de não serem indicadas vantagens em longo prazo.

## Indicações

Indicado como terapia adjuvante para o tratamento de OA, principalmente em relação à dor.

## Proloterapia de dextrose

O princípio fundamental da proloterapia de dextrose é a injeção de pequenos volumes de uma solução irritante em áreas de inserções dolorosas (ligamentos, tendões e em espaços articulares adjacentes), ao longo de várias sessões de tratamento. O mecanismo de ação ainda não é claro, mas hipóteses sugerem que a proloterapia estimula a cicatrização local de tecido extra e intra-articular cronicamente lesado, embora faltem evidências definitivas. Segundo a literatura, 51 as injeções de proloterapia podem ser adequadas para tratar a causa multifatorial da dor no joelho por OA.

## Indicações

Indicada como terapia adjuvante para o tratamento de OA, principalmente em relação à dor, mas atualmente não existem evidências para esse tratamento.

## Bloqueio dos Nervos Geniculares

No levantamento de literatura, foram encontrados sete estudos randomizados e apenas uma revisão sistemática.

O bloqueio do nervo genicular (BNG) é uma alternativa terapêutica para bloquear os três nervos sensoriais do joelho: os nervos genicular superior lateral e medial e o genicular inferior medial, o que faz com que a transmissão da dor para o sistema nervoso central seja impossibilitada, além de auxiliar na melhora das funções do indivíduo. 52 Esse bloqueio nervoso pode ser guiado por ultrassom, em que é possível visualizar um panorama direto das estruturas, o que permite um controle em tempo real da aplicação anestésica. 53 Assim, o bloqueio é realizado de forma mais eficiente, e gera menores latência e dependência de informações anatômicas, e utilização de menor quantidade de solução anestésica, além de proporcionar maior segurança. 54

Um estudo 54 foi desenvolvido com quatro pacientes idosos com OA de joelho em grau avançado, com limitação de movimentação e presença de dor crônica, submetidos ao BNG guiado por ultrassonografia. Os resultados demonstraram melhoras da dor e da capacidade funcional satisfatórias e importantes, não sendo observado nenhum tipo de complicação devido a esses bloqueios. 54 Uma comparação de eficiência entre a terapia de bloqueio intra-articular e a de BNG foi realizada mediante uma análise no período de doze semanas. 55 Ambas as técnicas denotaram resultados satisfatórios em relação à minimização de dor, à melhora na qualidade do sono noturno e ao desempenho de atividades diárias, mas a pesquisa destaca a utilização da técnica de BNG por representar uma alternativa de maior segurança, minimamente invasiva e de elevada eficiência. 55

## Indicações

Há evidências razoáveis para, pelo menos, direcionar o nervo genicular medial superior, o nervo genicular medial inferior e o nervo genicular medial inferior usando anestésicos locais, corticosteroides ou álcool para reduzir a dor e melhorar a função do joelho em pacientes com OA crônica do joelho sob orientação de ultrassom. O procedimento é seguro, mas mais pesquisas são necessárias para determinar a abordagem intervencionista ideal.

## Acupuntura

A acupuntura é uma técnica muito conhecida e utilizada em tratamentos de variados distúrbios por meio de inserção de agulhas finas em pontos anatômicos específicos do corpo do paciente. 56 A realização de estudos sobre a utilização da acupuntura para a minimização de dores provenientes de OA de joelho e reabilitação funcional vem aumentando, o que demonstra que essa terapia atua na mitigação dos sintomas pela ativação de variados elementos químicos dotados de bioatividade, por meio de mecanismos periféricos, espinhais e supraespinhais. 57

Teixeira et al. 6 avaliaram o tratamento de dois pacientes com OA do joelho mediante uma sessão semanal (durante seis semanas) de acupuntura, com sessões de trinta minutos de agulhamento em quatro pontos locais. Os pesquisadores 6 relataram minimização das dores e aumento da amplitude e da mobilidade de flexão articular do joelho dos pacientes, o que demonstra que a técnica é eficiente como tratamento alternativo ou complementar da OA, mesmo em curto espaço de tempo. De forma geral, a acupuntura pode propiciar uma melhora da percepção subjetiva da qualidade de vida do indivíduo com OA, o que permite um melhor desempenho das atividades diárias, mas fatores como o número e a duração das sessões de acupuntura, o estado de saúde do paciente e o tempo de prevalência da doença devem ser considerados.

## Indicações

Indicada como terapia para o tratamento de OA, principalmente em relação à dor.

## Prática de Exercícios Físicos

Diretrizes clínicas para o manejo da OA do joelho sugerem que os pacientes devem receber um conjunto básico de intervenções não farmacológicas, incluindo educação, perda de peso e exercícios de baixo impacto (fortalecimento, exercícios cardiovasculares e/ou exercícios mente-corpo, como ioga ou *tai chi* ). 58 Uma revisão sistemática recente 59 avaliou diferentes ECRs sobre a eficácia das atividades físicas não aquáticas para pacientes portadores de OA no joelho. Nesses estudos, os pacientes foram divididos em grupos submetidos a exercícios e grupos que não realizavam exercícios ou nenhum tratamento. Os autores 59 encontraram que, no grupo que realizou atividades físicas terrestres, houve redução consistente do quadro álgico e aprimoramento da forma física, o que melhorou sua qualidade de vida.

## Indicações

Pesquisas atuais 60 indicam que a prática de atividade aeróbica durante 150 minutos semanais com intensidade moderada, ou a realização de atividade física moderada/vigorosa, ou exercícios de fortalecimento muscular duas vezes por semana trazem muitos benefícios para indivíduos com OA do joelho preexistente.

## Outros

Outro recurso terapêutico é a palmilha de cunha lateral. Uma pesquisa recente 61 concluiu que órtese externa individualizada (fixada abaixo da sola) esteve associada a uma maior melhora na dor e no estado funcional do que uma órtese de controle. Da mesma maneira, órteses para a correção do varo ou do valgo, que atuam alterando a biomecânica, deslocando a carga para o compartimento menos comprometido, estão sendo implantadas como alternativas para ganho de função e melhora da dor.

## Considerações Finais

A OA do joelho é uma doença de alta prevalência e incapacitante. No decorrer dos últimos anos, muitas informações de grande importância sobre a causa e a patogênese da OA foram obtidas, o que pressagia uma nova era na terapêutica da OA. Entretanto, o manejo não deve ser generalizado, sendo necessária uma adequação a cada indivíduo, para que seja possível designar uma intervenção ideal para cada caso.

A mudança de estilo de vida, prática de exercício físico e realização de acupuntura, dentre outros, são métodos menos invasivos e indicados em primeira instância, e o procedimento cirúrgico deve ser deixado como alternativa para casos em que esses métodos não apresentaram respostas satisfatórias. Assim, a busca por medicamentos modificadores da doença para o tratamento da OA tornou-se uma prioridade no campo da ortopedia. O AH demonstrou ser seguro e eficaz no tratamento da dor relacionada à OA no joelho e em outras articulações, o que confirma que o AH pode ter alguma propriedade modificadora da doença.

Conclui-se, portanto, que mais estudos referentes a este tema devem ser realizados para que se possa alcançar um maior conhecimento científico e, por meio dele, compreender e preencher as lacunas ainda existentes.
